# Global Estimation and Mapping of the Conservation Status of Tree Species Using Artificial Intelligence

**DOI:** 10.3389/fpls.2022.839792

**Published:** 2022-04-29

**Authors:** Sandro Valerio Silva, Tobias Andermann, Alexander Zizka, Gregor Kozlowski, Daniele Silvestro

**Affiliations:** ^1^Department of Biology, University of Fribourg, Fribourg, Switzerland; ^2^Interfaculty Bioinformatics Unit, University of Bern, Bern, Switzerland; ^3^Global Gothenburg Biodiversity Centre, Department of Biological and Environmental Sciences, Sweden, University of Gothenburg, Gothenburg, Sweden; ^4^Department of Biology, Philipps-University Marburg, Marburg, Germany; ^5^Swiss Institute of Bioinformatics, Fribourg, Switzerland

**Keywords:** IUCN red list, neural network, R package, extinction risk, GBIF

## Abstract

Trees are fundamental for Earth’s biodiversity as primary producers and ecosystem engineers and are responsible for many of nature’s contributions to people. Yet, many tree species at present are threatened with extinction by human activities. Accurate identification of threatened tree species is necessary to quantify the current biodiversity crisis and to prioritize conservation efforts. However, the most comprehensive dataset of tree species extinction risk—the Red List of the International Union for the Conservation of Nature (IUCN RL)—lacks assessments for a substantial number of known tree species. The RL is based on a time-consuming expert-based assessment process, which hampers the inclusion of less-known species and the continued updating of extinction risk assessments. In this study, we used a computational pipeline to approximate RL extinction risk assessments for more than 21,000 tree species (leading to an overall assessment of 89% of all known tree species) using a supervised learning approach trained based on available IUCN RL assessments. We harvested the occurrence data for tree species worldwide from online databases, which we used with other publicly available data to design features characterizing the species’ geographic range, biome and climatic affinities, and exposure to human footprint. We trained deep neural network models to predict their conservation status, based on these features. We estimated 43% of the assessed tree species to be threatened with extinction and found taxonomic and geographic heterogeneities in the distribution of threatened species. The results are consistent with the recent estimates by the Global Tree Assessment initiative, indicating that our approach provides robust and time-efficient approximations of species’ IUCN RL extinction risk assessments.

## Introduction

Of the estimated 350,000 vascular plant species, the c. 59,000 described trees species (Botanic Gardens Conservation International, BGCI; Beech et al., 2017) represent the bulk of biomass and are essential as ecosystem engineers housing and feeding millions of species (Olson et al., 2001; Crowther et al., 2015; Bar-On et al., 2018). Furthermore, trees provide many of nature’s contributions to people supporting the livelihood of virtually all humans, e.g., as sources of wood, food, shade, firewood, construction, and carbon sinks (Luyssaert et al., 2008; Fazan et al., 2020; Davies et al., 2021.

However, an increasing proportion of tree species are subject to anthropogenic threats. The global IUCN RL, arguably the most influential and comprehensive framework to estimate species risk with extinction, lists 67 tree species that are either extinct (EX) or extinct in the wild (EW) (IUCN, 2021). Many more species (*n* = 11,548) are currently listed as threatened with extinction meaning that they have been classified by experts to face extremely high to high risk of extinction in the wild, based primarily on criteria related to range size, population size, or population decline.

The RL provides detailed and verified information on species extinction risk and the potential threats; and is the basis for conservation policy [informing, for instance, e.g., Convention on Biological Diversity (CBD), Intergovernmental Science-Policy Platform on Biodiversity and Ecosystem Services (IPBES), and Convention on International Trade in Endangered Species (CITES)] and prioritization of millions of dollars of conservation funds worldwide. Yet, due to the standardized criteria, the expert-focused assessment process, and the required documentation, the IUCN RL assessments are data intensive and time-consuming (Juffe-Bignoli et al., 2016; IUCN Standards and Petitions Subcommittee, 2017), and therefore, the IUCN RL is taxonomically and geographically biased (Bachman et al., 2019), and many species are classified as data deficient (DD) or have not been evaluated (Nic Lughadha et al., 2020; IUCN, 2021). Hence, until early 2021, c. 30,000 tree species remained without an RL assessment, forcing conservation decisions based on incomplete and biased information. Furthermore, for efficient conservation measures, extinction risk assessments need to be repeated and updated regularly. Keeping extinction risk assessments up to date is a major challenge for the IUCN RL (Rondinini et al., 2014), because, in addition to the general data and time constraints of RL assessments, reassessments of already red-listed species are often less appealing and hence down-prioritized.

As an attempt to speed up the process of assessing species extinction risk for the RL, a variety of methods have been proposed to automatically approximate species extinction risk based on species occurrence data from online databases (Bachman et al., 2011; Dauby et al., 2017; Pelletier et al., 2018; Zizka et al., 2021a, see Cazalis et al., 2022 for a review). All these methods have important caveats (Rivers et al., 2011; Nic Lughadha et al., 2019; Walker et al., 2020) and cannot replace the rigorous RL assessments. Yet, they can provide an approximation of species extinction risk and offer the advantage of being scalable to potentially large number of species.

For trees, a separate effort to boost the proportion of species with known extinction risk assessment exists: the Global Tree Assessment (GTA; BGCI, 2021). The GTA aims to assess the conservation status of all tree species following IUCN RL criteria to allow effective prioritization of conservation measures (Newton et al., 2015). As of late 2021, the GTA included, approximately 43,700 species, with about 20% of the known species yet to be assessed or classified (BGCI, 20211). To achieve this remarkable result, the GTA used a combination of semi-automated assessments based on approximate species range size from available occurrence data (for approximately 10,000 species) and data from national assessments and new expert-based assessment. The process involved the coordination of a huge international effort that took 5 years of research involving 60 institutional partners and over 500 experts (BGCI, 2021), which exemplifies the complexity of this approach. In contrast to the IUCN RL, the GTA identified 142 tree species as EW.

In this study, we present an automated assessment of the extinction risk of all tree species for which occurrence data are available at the Global Biodiversity Information Facility (The Global Biodiversity Information Facility [GBIF], 2021). Building upon the recently published R package IUCNN (Zizka et al., 2022a), we harvested and preprocessed the occurrence data of tree species already assessed in the IUCN RL and trained a deep learning model to infer the extinction risk status of tree species not yet assessed on the RL. Furthermore, we used the resulting assessments with geographic distribution and threat level, to highlight the most threatened taxonomic groups and to identify the biomes and countries most vulnerable to anthropogenic pressure. We demonstrated the reliability of our estimates by measuring the prediction accuracy and its spatial consistency.

## Materials and Methods

### Data Collection and Preprocessing

We obtained the most recent database of scientific names of tree species from GlobalTreeSearch (Beech et al., 2017, version 1.5), which included 58,496 species. We retrieved the IUCN Red List extinction risk category using the R package rredlist (Chamberlain, 2020; R Core Team, 2021) from www.iucnredlist.org, yielding red list categories for 32,899 species (retrieved on 3 October 2021). This also included the categories data deficient (DD) (2,332 species) and EX as well as EW (together 67 species). For the purpose of training a supervised learning model, we disaggregated the data into 5 classes of interest, namely, least concerned (LC), near threatened (NT), vulnerable (VU), endangered (EN), and critically endangered (CE), which totaled 30,500 species.

We then retrieved occurrence data from the Global Biodiversity Information Facility^1^ using the R libraries taxize and rgbif (Chamberlain et al., 2020, 2021). The search returned 47,626,060 records (retrieved on 21.09.2021; DOIs in Supplementary Table 1; see also Supplementary Data). Since species occurrence records from the public database are error prone (Maldonado et al., 2015; Zizka et al., 2020), we cleaned the raw occurrences in a series of automated steps. First, we removed records that could not be assigned to a species from our initial list, for instance, due to synonymy (Cayuela et al., 2012; TPL v1.0). Second, we removed duplicates and retained only records derived from human observation, and preserved specimens or literature, with a coordinate uncertainty smaller than 100 km. Finally, we used the R package CoordinateCleaner v.2.0-20 (Zizka et al., 2019) to remove occurrences with suspicious coordinates falling into a capital city, country centroids, the GBIF headquarter, known biodiversity institutions, the sea, the point 0/0, if the latitude and longitude were equal, or if the occurrence was detected as a spatial outlier. After these cleaning steps, our dataset included 23,535,210 occurrences from 49,743 species.

### Feature Generation

We used the IUCNN R package (Zizka et al., 2022a) to calculate features for each species based on their occurrences. The extracted features included geographic information (i.e., number of occurrences, area of occurrence, extent of occurrence, and latitudinal range), presence of the species across different biomes, proxies for climate, and human footprint (all features are described in Supplementary Table 1 in Zizka et al., 2021a). In some cases, not all features could be calculated, and we omitted those species because the downstream IUCNN functions cannot currently handle incomplete feature sets. The final dataset included features and IUCN RL labels for training for 27,146 species and features only for 21,691 species (for which we estimated extinction risk).

### Model Training

The package IUCNN provides a framework to access the Python library Tensorflow (Abadi et al., 2016) within R. Using this framework, we trained fully connected neural networks with fivefold cross-validation to estimate the prediction accuracy across all samples. In each fold, the data were split into 80% of the instances used for training and 20% for validation. We monitored validation loss during training as a stopping rule to prevent overfitting. We then computed the prediction accuracy, quantifying the expected performance of the model on unseen data as an average of the validation accuracy across the fivefolds. After preliminary tests, we set the architecture of the neural network to three hidden layers with 100, 60, and 20 nodes, respectively, and rectifier linear unit (ReLU) activation functions. Using the IUCNN implementation, we tested two neural network models, a classifier with a SoftMax activation function in the output layer and a regression model. Furthermore, we used dropout (Gal and Ghahramani, 2016) with the rate set to 0.1 to prevent overfitting and allow the estimation of prediction uncertainty. We trained the networks based on the five extinction risk classes and using a simplified binary classification, including possibly threatened (i.e., VU, EN, and CR) and possibly not threatened (i.e., LC and NT), which we shortened to “not threatened” hereafter. We evaluated the performance of the models using the cross-validation accuracy.

### Predicting Species Conservation Assessment

We used the trained models to predict the extinction risk of the 21,691 unlabeled species in our dataset. The application of 100 Monte Carlo (MC) dropout replicates allowed us to measure uncertainty around predictions (Gal and Ghahramani, 2016). We combined our predictions with the available RL assessments to summarize the estimated extinction risk within higher taxa and by region. Specifically, we computed the number and proportion of threatened species in each family to quantify the level of heterogeneity in conservation status among taxonomic groups. After assigning species to countries and biomes, we also computed the number and proportion of threatened species within these spatial entities.

### Sensitivity Tests

We performed sensitivity tests to assess the extent of taxonomic and geographic bias among the species used for training our models, i.e., the species in the RL. Specifically, we looked at the fraction of evaluated species across plant genera, families, and orders assuming that a systematic bias would leave a signature in their distribution. For instance, if the assessments were carried out systematically by the taxonomic group, we would expect a bimodal distribution where the fraction of assessed species nears one in some groups and zero in others. Similarly, we quantified the fraction of assessed species across countries and biomes to estimate the level of heterogeneity in the available RL assessments.

We then calculated the cross-validation prediction accuracy for each country, to evaluate whether spatial biases in the distribution of RL-assessed species may impact the accuracy of our predictions. Specifically, we identified what tree species in our test sets (from the 5 cross-validation folds) were found in each country, based on the available geographic occurrences. We then approximated the prediction accuracy for each country as the fraction of species correctly classified out of all tree species occurring in the country.

We performed 100 predictions for all species using MC dropout probability as a measure of uncertainty around each prediction (Gal and Ghahramani, 2016). This enabled us to identify the MC dropout probability above which the classified instances yield a predefined prediction accuracy. For instance, we could identify the MC dropout probability threshold such that instances classified with a higher probability yield a 95% test accuracy, while others will remain “unclassified” (Gal and Ghahramani, 2016). We performed this test to assess how many species could be classified with high confidence (accuracy > 90%) and whether the fraction of them assigned to the possibly threatened category changes compared with the full set of predicted species.

## Results

### Model Selection and Performance of the Best Models

The best-fitting model for the 5-class prediction was a neural network classifier, which achieved a cross-validation prediction accuracy of 66.9%, while the regression model yielded an accuracy of 61.5%. LC species were correctly identified in 92.6% of the cases, while the accuracy was lower for the other classes, particularly, the intermediate NT and VU classes (Supplementary Figure 1). In most cases, the misclassified species were assigned to a neighboring class, indicating that the model could still correctly identify some signal for these species.

**FIGURE 1:**
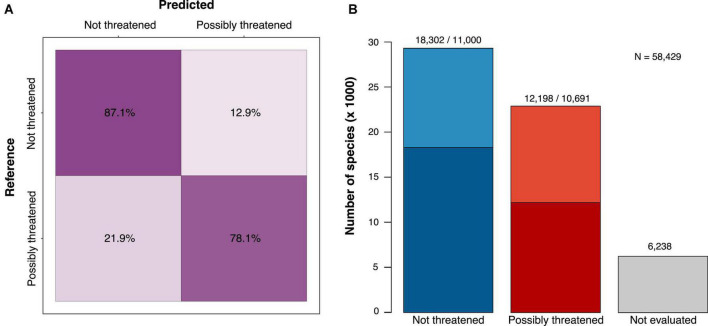
The classification results and performance of a deep learning model trained to identify possibly threatened tree species (binary classification; see Supplementary Figure 1 for the 5-class confusion matrix). **(A)** Confusion matrix showing the per-class prediction accuracy (cross-validation test sets) and **(B)** assessment of 58,429 species combining the IUCN RL (darker shades) and our predictions (lighter shades). Of the 52,191 species that could be assessed, 56% were estimated as not threatened and 44% as possibly threatened.

The best model for the 2-class prediction (not threatened vs. possibly threatened) was a neural network classifier, which achieved a prediction accuracy of 83.7%, similar to the corresponding regression model (83.5%). The accuracy was much more balanced among assessments than in the model with 5 classes, with 87.1% of the not-threatened species and 78.1% of the possibly threatened species correctly classified (Figure 1A). Given the substantially higher accuracy of the binary predictions, we focused, hereafter, on the results obtained from this model.

### Classifications

Our model predictions resulted in an increase in the number of extinction risk assessments from 30,500 to 52,191 species compared to the RL (the detailed classification results are available in Supplementary Table 2), reducing the number of tree species without either a full IUCN assessment or a preliminary automated assessment from 29,462 to 6,238 (Table 1). They remained unassessed because the features could not be generated for these species due to a lack of occurrence data.

**TABLE 1:** The number of tree species in different extinction risk categories on the official IUCN RL and following predictions by our deep learning approach.

Category	IUCN RL	%	Predictions	%	merged	%
5 classes						
LC	16,349	53.6	11,670	58.0	28,019	53.7
NT	1,953	6.4	4	0.1	1,957	3.7
VU	4,864	15.9	3,569	14.4	8,433	16.2
EN	4,836	15.9	4,248	20.4	9,084	17.4
CR	2,498	8.2	2,200	7.1	4,698	9.0
NE/DD	27,929		6,238		6,238	
2 classes						
Not threatened	18,302	60.0	11,000	50.7	29,302	56.1
Possibly threatened	12,198	40.0	10,691	49.3	22,889	43.9

With the binary classification model, we predicted 50% of the species as not threatened, while the Red List assessments consist of 60% not-threatened species (Table 1). Thus, we added more possibly threatened species than could be expected by extrapolating from the RL existing frequencies. We estimated 22,889 tree species as possibly threatened (39.1%) and 29,302 as not threatened (50.1%, Figure 1B and Table 1).

### Taxonomic Patterns of Tree Conservation Status

**FIGURE 2:**
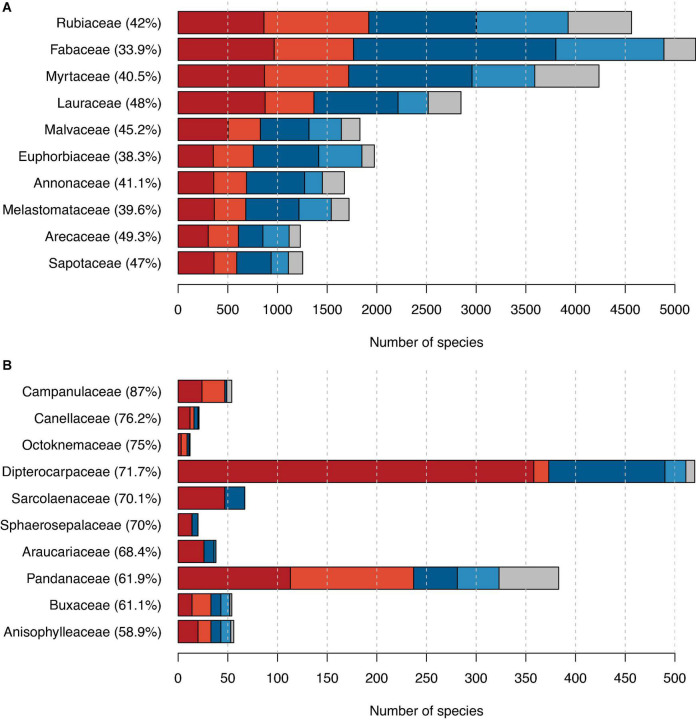
The proportion of possibly threatened species among trees grouped by families. **(A)** The 10 families with the highest number of possibly threatened tree species and **(B)** the 10 families with the highest proportion of possibly threatened tree species (and comprising more than 10 tree species in total). Red indicates counts of possibly threatened species, and blue indicates counts of possibly not threatened species, with darker shades used for IUCN RL assessments and lighter shades for our automated assessments. In gray, we showed the number of species not assessed. Percentages next to family names indicate the percentage of possibly threatened tree species in this family.

Our dataset included 288 families and 57 orders with tree species (Supplementary Tables 3, 4), several of which we estimated to include a large fraction of possibly threatened species. The family with the highest number of possibly threatened tree species was the Rubiaceae (Figure 2A). With its 4,838 tree species of which 3,925 were assessed in this study, it is the second most species-rich family in terms of tree species. The family with the most tree species was the Fabaceae with a total of 5,483 tree species of which, we assessed 4,890, among these 1,765 as possibly threatened. In 13 families, the percentage of threatened tree species was 100%; however, those were all families comprising only 1–3 species (Supplementary Table 3). When considering only families with more than 10 evaluated tree species, Campanulaceae had the highest proportion of possibly threatened species (87%; Figure 2B).

### Spatial Patterns of Tree Extinction Risk

**FIGURE 3:**
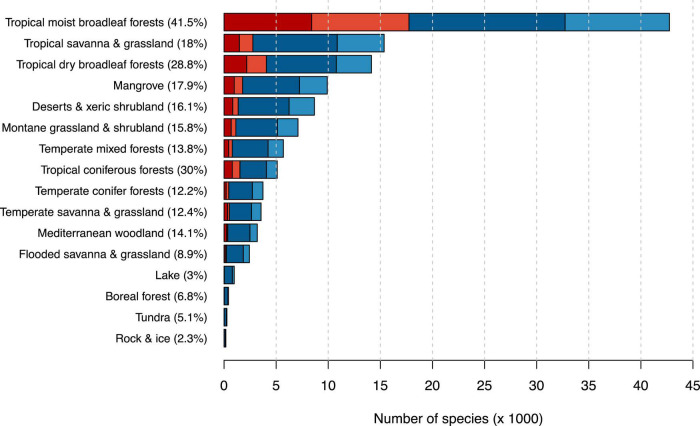
Number of possibly threatened (red) and not threatened (blue) tree species in different biomes after Olson et al. (2001). Darker shades indicate species count from the IUCN RL, while lighter shades indicate species counts from our automated assessment. Percentages next to biome names indicate the percentage of threatened tree species in this biome. Biome names are simplified for better readability.

With more than 42,000 tree species, the tropical moist broadleaf forest was the most diverse biome in our dataset (Figure 3 and Supplementary Table 5). It also comprised the highest number of possibly threatened species (17,749), meaning that we estimated 41.5% of all tree species occurring in tropical moist broadleaf forests to be possibly threatened. The second highest fraction of possibly threatened tree species occurred in tropical coniferous forest comprising 5,107 tree species with 1,530 of them (30.0%) predicted to be possibly threatened.

**FIGURE 4:**
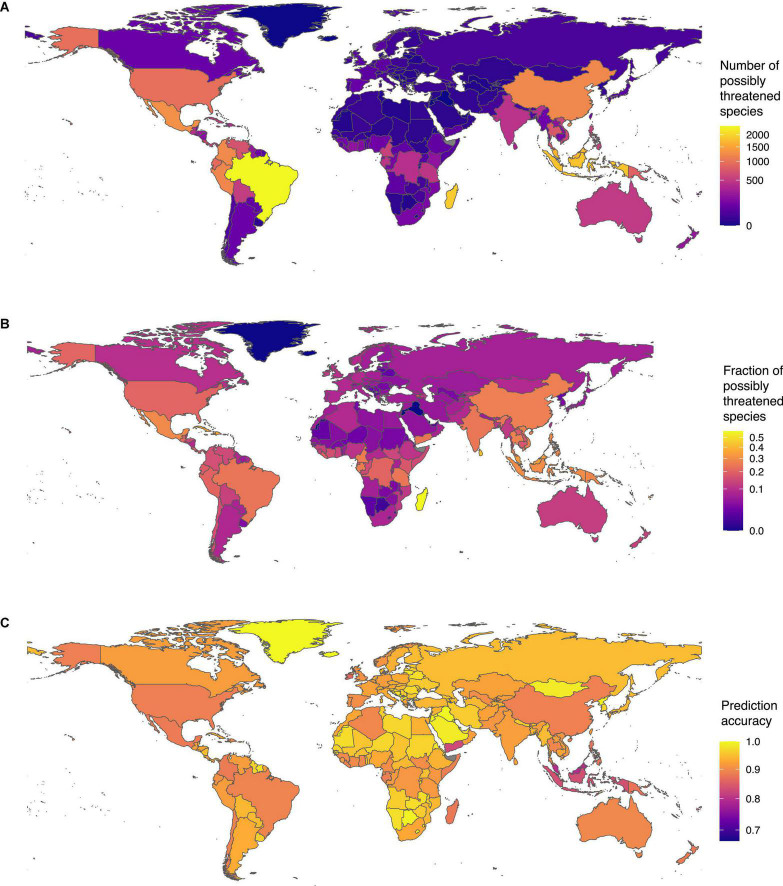
Tree species threat level around the globe: **(A)** number of threatened tree species per country and **(B)** fraction of threatened tree species per country (with at least 5 assessed species). **(C)** Prediction accuracy across countries: Despite the spatial biases in IUCN RL assessments, our model performed well with estimated accuracy above 80% in most countries.

We found the highest sampled diversity of tree species in our dataset in Brazil (Supplementary Table 6). The country harbored 9,995 species, of which, 2,397 were possibly threatened, making Brazil also the country with the highest number of possibly threatened species (Figure 4A). The fraction of possibly threatened tree species was highest in Madagascar. Including our status predictions, we have extinction risk information for almost all tree species in Madagascar (3,332 of 3,335). Of these tree species, we found 57%, (*N* = 1,893) as possibly threatened (Figure 4B).

### Sensitivity Tests

A taxonomic bias in the subset of species that have already been evaluated by the IUCN and which we used to train our models could hamper the accuracy of our supervised learning method on other species. The fractions of IUCN Red List evaluated species within orders, families, and genera followed unimodal distributions except for a slight over-representation of fully assessed groups (Supplementary Figure 2). This suggests a lack of systematic bias in the evaluated data, despite their variability across taxonomic groups. We observed a similar pattern among all species and among the subset of 48,571 species that were used in this study (fewer species due to cleaning steps). Additionally, the IUCN assessment rates among orders, families, and genera were independent of the number of species they encompassed, thus pointing to a lack of systematic bias in the training data (Supplementary Figure 2).

Ignoring countries comprising fewer than 10 tree species, all countries had at least 40% of their tree species assessed on the RL, and these fractions of assessed species across countries followed a unimodal distribution (95% range: 45.6–87.9%; Supplementary Figure 3A). We did however observe a trend toward higher assessment fractions in countries with fewer tree species Supplementary Figure 3B). Similarly, assessment fractions across biomes showed a trend toward lower assessment fractions in highly diverse biomes (Supplementary Figure 3C). For instance, in boreal forests, 83% of trees were assessed on the RL, while in tropical moist broadleaf forests, only 52% of all tree species were assessed.

Despite the heterogeneous fraction of assessed species in the RL across countries (Supplementary Figure 3C), the prediction accuracy of our model was high and relatively homogenous across countries (Figure 4C). For instance, in most species-rich countries in South America and Central Africa, the prediction accuracy exceeded 80% despite a generally lower fraction of species included in the RL. The prediction accuracy was however slightly lower in Southeast Asia, where the fraction of species evaluated on the RL is lowest. Overall, these results show that our model managed to capture the general properties of species conservation status without overfitting toward regions of the world with denser data.

Limiting the predictions to those with a higher MC dropout probability, yielding a prediction accuracy of 90%, reduced the number of species that could be confidently assessed to 16,703, thus leaving 4,988 species unclassified. However, the proportion of possibly threatened species among the evaluated species remained similar, slightly decreasing from 46.9 to 45.1% (Supplementary Table 2). This indicates that while slightly more species in the not-threatened class could be predicted with high confidence compared with that in possibly threatened species, the results are robust to prediction uncertainty.

## Discussion

Improved knowledge of species extinction risk helps to guide conservation effort and avoid taxonomically and geographically biased decisions. Trees are pivotal to human livelihood and play a fundamental role in most terrestrial ecosystems (Chavan et al., 2016; Watson et al., 2018; Keppel et al., 2021). Thus, in some cases, a focus of conservation efforts on protecting tree species and, hence forests, can be an effective way to conserve a large share of biodiversity (Watson et al., 2018). This importance of trees is one reason why evergreen rainforests have long been at the forefront of conservation effort at the expense of other diverse and unique habitats (Parr et al., 2014; Veldman et al., 2019; Silveira et al., 2021). The ecological and economic importance of trees and their potential as umbrella species are reasons for the concentrated effort and systematic assessment of the GTA in 2015 (Newton et al., 2015). In this study, we complemented this effort with an automated deep learning assessment to approximate extinction risk assessments for all tree species with sufficient distribution data available, within a fraction of the time needed for full assessments on the RL or during GTA. Our results show that thousands of tree species are possibly threatened with extinction and that their state of conservation is heterogeneous among taxonomic groups and across different biomes and countries.

Using machine leaning approaches is increasingly common in biological research, for instance, to infer the intraspecific genetic diversity of amphibian taxa or predict the conservation status of data-deficient mammals (Bland et al., 2014; Barrow et al., 2020; Lee et al., 2020). More specifically, using machine learning to assist conservation prioritization is an active field of development (Walker et al., 2020; Cazalis et al., 2022; Silvestro et al., 2022; Zizka et al., 2022a), and automated methods have the potential to process large numbers of species quickly (Pelletier et al., 2018; Zizka et al., 2021b).

The increased assessment speed together with the capacity to close taxonomic gaps of knowledge by transferring knowledge from groups with good data availability (i.e., the training data) is a clear strength of automated assessment methods (Cazalis et al., 2022). Yet, automated methods to approximate extinction risk face several challenges in the IUCN RL framework, including minimum data requirements (Rivers et al., 2011; Nic Lughadha et al., 2019), inability to explicitly use the IUCN criteria and subcriteria, and a reduced documentation (which is why most automated assessments cannot feed back into the IUCN RL, Cazalis et al., 2022), as well as the error rate and low traceability and transparency (Walker et al., 2020). The IUCNN approach has the specific strength that it can integrate heterogeneous input features, yet it is sensitive to the class imbalance in the training data and prone to underestimating the number of species in intermediate extinction risk categories, when using the full suite of IUCN categories (see Zizka et al., 2022a for details). Automated assessments are complementary to full assessments on the IUCN RL. We consider filling knowledge gaps of extinction risk in specific taxonomic groups or geographic regions for the use in (1) synthetic academic research (for instance, linking extinction risk to species functional traits), (2) conservation communication to a broader public (for instance, indicating possibly threatened species in an ecosystem), and (3) conservation research (for instance, identifying priority species for full IUCN assessments), the prime applications for automated assessments.

While species occurrence data from large databases are inevitably affected by error, previous studies (Walker et al., 2020, 2021; Zizka et al., 2020) have shown that using stricter filters to select the species occurrences (for instance, by only considering recent occurrence records when generating the features) did not substantially increase the predictive accuracy but did decrease substantially the number of species that can be predicted (Zizka et al., 2020). We, therefore, opted to limit record cleaning to basic automated filters.

Our predictions are based on a range of data that can be obtained from publicly available geographic occurrence records. In contrast to Pelletier et al. (2018) who included morphological trait data such as woodiness, leaf phenology, and plant height to predict plant conservation status, we focused on data derived from geographic occurrences exclusively. We assumed that, since “trees” are a functionally defined group [i.e., woody, tall, few, or single-stemmed, as defined by Botanic Gardens Conservation International (BGCI); Beech et al., 2017], the variation in traits for which data were available (mostly records of traits such as growth form, maximum height, etc.) was negligible. Thus, we considered these traits uninformative in our case. However, we acknowledge that the inclusion of additional data (such as species economic value and human use) might contribute to improving the predictions. To include anthropogenic factors in the model, we used the human footprint in areas of occurrence of the species, as suggested by previous studies (Venter et al., 2016; Walker et al., 2020).

One of the main concerns in using supervised learning methods such as neural networks is the imbalanced representation of classes in the training set. This is an inevitable property of data from the IUCN RL, where some classes, e.g., LC, are over-represented compared with others, e.g., VU. Pelletier et al. (2018) addressed this issue, in a random forest model, by sub-sampling the training data. This, however, means excluding training instances, i.e., discarding available information, to obtain a more balanced training dataset. In this study, we tackled this issue by grouping the five IUCN classes into the two broader “possibly threatened” and “not threatened” classes. The resulting binary classification, resulted in more balanced classes and higher prediction accuracy, as expected based on previous studies (Stévart et al., 2019; Zizka et al., 2020). Still, non-negligible error remains in identifying species extinction risk and, in our model, classifying as not threatened the several species that appear as threatened on the IUCN RL. While some of the error is linked to the imperfection of predictive methods, we also found that several misclassified species include cultivated plants. These include the Fraser fir (*Abies fraseri*), several *Araucaria* species, *Ginkgo biloba, Sequoia sempervirens*, and *Magnolia stellata*, all of which are at risk of extinction in the wild, but widely found in cultivated settings and gardens. Their wide distribution as cultivated plants is reflected in their recorded geographic occurrences and probably explains the discrepancy between RL and automated assessments.

Since we applied a supervised learning approach, it is important for the data to be unbiased meaning that the assessed species should not differ systematically from unassessed species. We performed several tests to detect possible biases in the data and found no evidence of systematic taxonomic biases, with most groups sharing similar fraction of species already included in the RL. We did, however, detect evidence of geographic bias, with some regions of the world (especially, highly diverse tropical areas) presenting a lower fraction of assessed species in the RL. Yet, the estimated accuracy of our model was relatively unaffected by this bias, showing that the predictions obtained through the neural network were robust to this bias.

The number of possibly threatened species per plant family correlated with the overall number of species per family: The ten plant families with most possibly threatened species (Figure 2A) were also the ten families with the highest overall tree richness and represent some of the most species-rich families worldwide (Ehrendorfer et al., 2018). In contrast, the list of families with the highest fraction of possibly threatened species (Figure 2B) comprises different families with likely individual reasons for the high proportion of possibly threatend species. For six out of ten families in this list, a specific common threat related to a globally restricted distribution seems plausible. The families Sphaerosepalaceae and Sarcolaenaceae are endemic to Madagascar, and in the Buxaceae and Pandanaceae, the most species-rich genera *Buxus* and *Pandanus*, respectively, have centers of diversity and endemism in Madagascar. Hence, the high extinction risk in these families is likely linked to the high rates of land use change in Madagascar and their known detrimental effect on biodiversity. The possibly threatened tree species in Campanulaceae belonged exclusively to the peculiar radiation of phylogenetically derived woody Campanulaceae species on the Hawaiian archipelago (genera *Clermontia*, *Cyanea*, *Delissea*, *Sclerothec*a, and *Trematolobelia*, Givnish et al., 2009; Zizka et al., 2022b). Hence, the high proportion of threatened tree species in Campanulaceae is linked to the low proportion of tree species in the family (most Campanulaceae are herbaceous) and the specific threat by land use change and invasive plants and animals in the Hawaiian archipelago. The gymnosperm family Araucariaceae mostly comprises species in the genera *Araucaria* and *Agathis* occurring in Australasia and South America, which are often threatened by logging and human fire suppression;^2^ plus the “*living fossil*” *Wollemia nobilis*, only known from New South Wales in Australia. In Canellaceae, Octoknemaceae, and Anisophylleacea, the reasons for the high proportion of threatened species are less clear since these families have a larger geographic distribution. Yet, species in these families are mostly forest species in tropical and subtropical Africa and America threatened by on-going land use change. Similarly, Dipterocarpaceae are important elements of tropical rainforests particularly in Southeast Asia often threatened by logging and deforestation.

Since the tropical moist broadleaf forest comprises most known tree species, it unsurprisingly also harbors the highest number of threatened trees. However, in this biome, we also predicted 41.5% of the tree species to be threatened with extinction, while no other biome exceeds 30%. We considered mostly two reasons for the outstandingly high number of possibly threatened species in the tropical moist broadleaf forest. First, many tropical and subtropical islands harbor tropical forests with high numbers of endemic species and high levels of anthropogenic threat at the same time, for instance, New Caledonia, the Philippines, and Madagascar (Mittermeier et al., 1996; Myers, 1988). These biodiversity hotspots are unique because they harbor many endemic plant and animal species and face high rates of depletion. Second, tree species in the moist tropical forest often have small populations (for instance, less than 1,000 individuals for an estimated 6,000 tree species in Amazonia; ter Steege et al., 2013), and individuals are often scattered throughout their range (Zizka et al., 2018). Small population sizes and the resulting small area of occupancy are likely to lead to an increased species risk of extinction and are explicit criteria in RL assessments. In contrast, average range sizes of trees in other biomes, for instance, in African savannas or boreal forests, are often large.

Countries with tropical forests show high numbers and fractions of possibly threatened tree species, in line with our observation that tropical biodiversity hotspots are exposed to high risks. The spatial patterns of extinction risk estimated in this study are consistent with the estimates produced independently by the GTA (BGCI, 2021), indicating that they are robust and not a product of the data or method used. Brazil harbors the highest number of threatened species, and the threat is continuing as the timber of possibly threatened species is traded in vast amounts, primarily to countries of the global North (Brandes et al., 2020), and the rates of deforestation remain extremely high (accessed on 13. August, 2021).^3^ The high proportion of possibly threatened species in Madagascar is consistent with the recent report of Beech et al. (2021), which estimated 63% of Malagasy species to be threatened. Among them, there are many Pandanaceae species (Callmander et al., 2007), 3 of the 6 endemic baobab (*Adansonia* spp., Malvaceae), and the newly described Sapotaceae species *Labramia ambondrombeensis* (Baum, 1995; Randriarisoa et al., 2020). As 93% of Madagascar’s tree species are endemic, conservation efforts in the country are fundamental to preserve this staggering and unique diversity (Beech et al., 2021) and conserve the basis for a sustainable development of the countries’ human population.

Trees represent an irreplaceable component in most terrestrial ecosystems, and the very existence of entire biomes depends on their biodiversity. We found that a large fraction of all tree species are at risk of extinction, and available data show that extinctions have already taken place in recent years. Yet, it is not too late to prevent the loss of most of the tree biodiversity, but conservation efforts must step up now. We hope that our results can help prioritizing conservation action and raising awareness of the urgency to address the ongoing biodiversity crisis.

## Data Availability Statement

The original contributions presented in the study are included in the article/Supplementary Material; further inquiries can be directed to the corresponding author/s. Supplementary codes and data are available in a Zenodo repository with Doi: 10.5281/zenodo.5195786.

## Author Contributions

SS, GK, and DS designed the study. SS, TA, and DS performed the analyses. SS, AZ, GK, and DS wrote the manuscript. All authors contributed to the article and approved the submitted version.

## Conflict of Interest

The authors declare that the research was conducted in the absence of any commercial or financial relationships that could be construed as a potential conflict of interest.

## Publisher’s Note

All claims expressed in this article are solely those of the authors and do not necessarily represent those of their affiliated organizations, or those of the publisher, the editors and the reviewers. Any product that may be evaluated in this article, or claim that may be made by its manufacturer, is not guaranteed or endorsed by the publisher.

## Footnotes

1
www.gbif.org

2
www.iucnredlist.org

3
globalforestwatch.org
