# Supplementary material for: Global Estimation and Mapping of the Conservation Status of Tree Species Using Artificial Intelligence
Source: Front Plant Sci. 2022 Apr 29;13:839792. doi: 10.3389/fpls.2022.839792 (PMC9100559; doi:10.3389/fpls.2022.839792)
Supplement: Supplementary file 1 [file Presentation_1.pdf]

SUPPLEMENTARY FIGURES

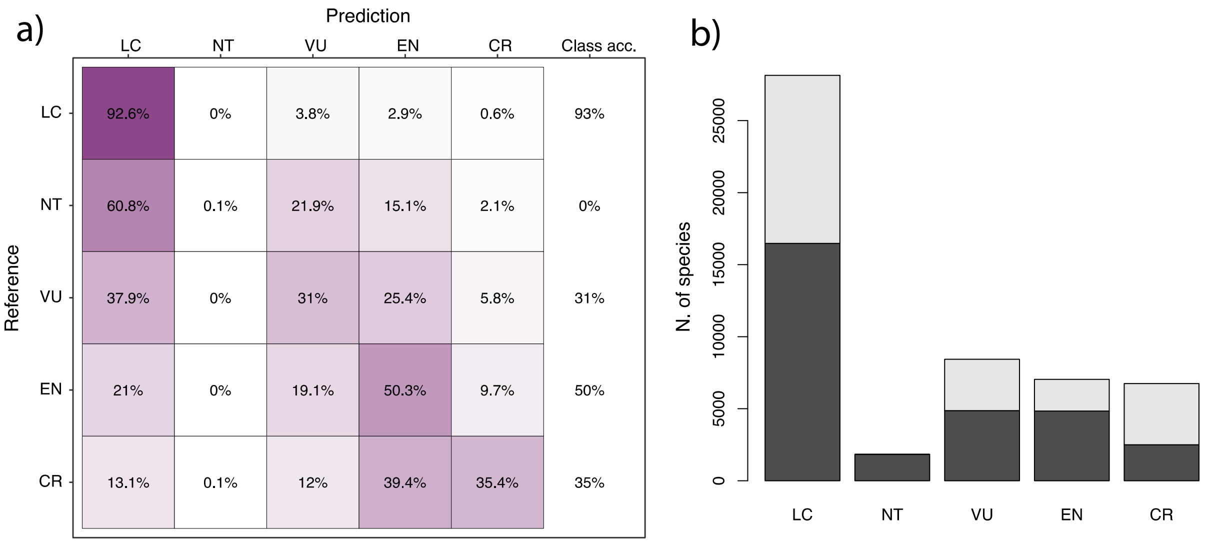

Figure S1. Results from our neural network classifier with 5 classes. a) confusion matrix showing the cross-validation accuracy and b) distribution of the 5 labels in the RL (dark grey) and in our predictions (light grey). The full results are reported in Tables S2.

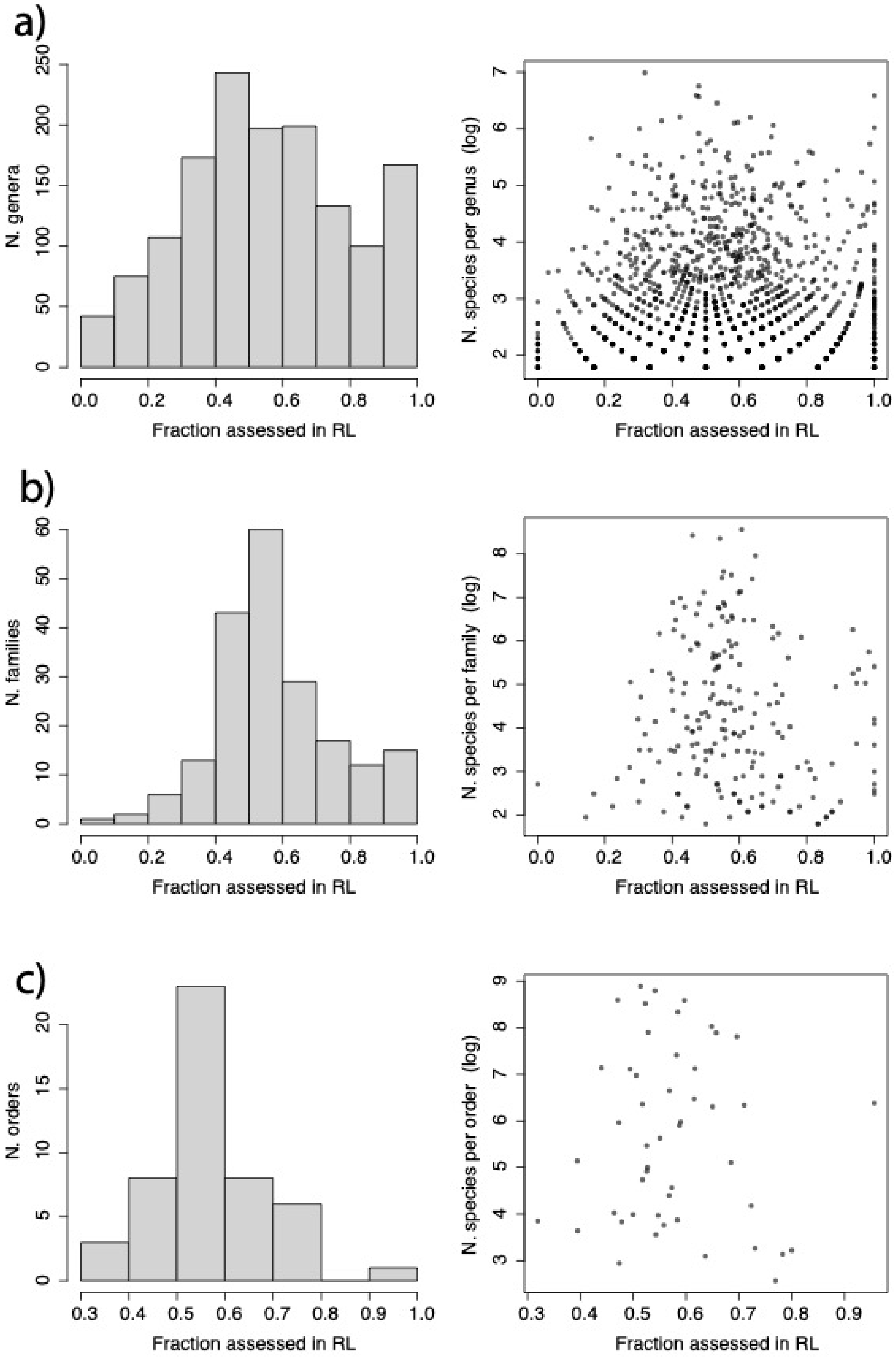

Figure S2. Fraction of assessed tree species in the RL across genera (a), families (b), and orders (c). In the scatterplots the fraction of assessed species is plotted against the log-transformed number of species across taxonomic groups.

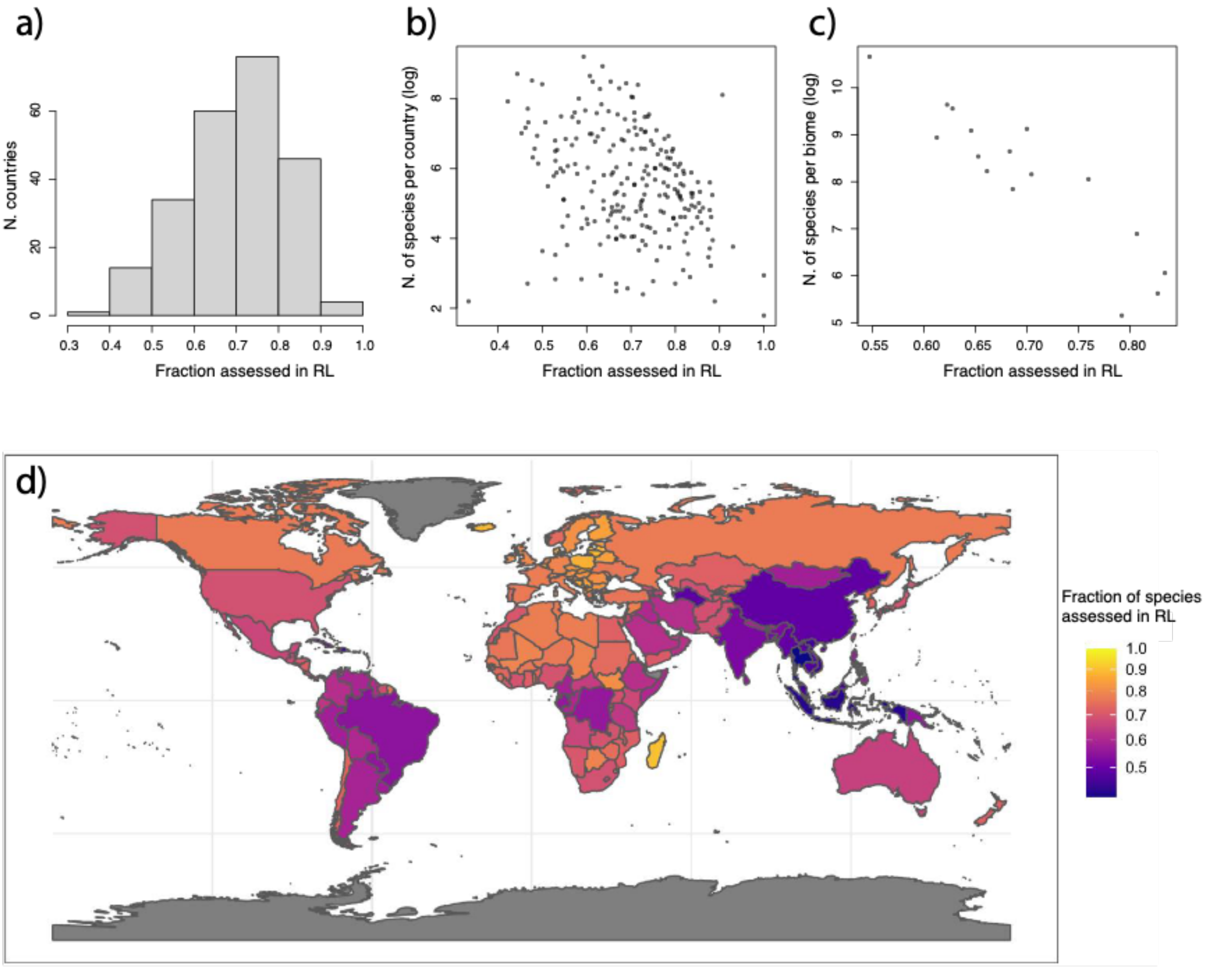

Figure S3. Fraction of assessed tree species in the RL across countries (a, b, d) and biomes (c). Scatter plots in b) and c) show a negative correlation between the species richness of a country or biome and the fraction of species assessed in the RL.
